# The Vitamin D Analog, MART-10, Attenuates Triple Negative Breast Cancer Cells Metastatic Potential

**DOI:** 10.3390/ijms17040606

**Published:** 2016-04-21

**Authors:** Kun-Chun Chiang, Ta-Sen Yeh, Shin-Cheh Chen, Jong-Hwei S. Pang, Chun-Nan Yeh, Jun-Te Hsu, Li-Wei Chen, Sheng-Fong Kuo, Masashi Takano, Atsushi Kittaka, Tai C. Chen, Chi-Chin Sun, Horng-Heng Juang

**Affiliations:** 1 General Surgery Department and Zebrafish Center, Chang Gung Memorial Hospital, Chang Gung University, Keelung 20401, Taiwan; 2 General Surgery Department, Chang Gung Memorial Hospital, Chang Gung University, Taoyuan 20401, Taiwan; tsy471027@adm.cgmh.org.tw (T.-S.Y.); chensc@adm.cgmh.org.tw (S.-C.C.); yehchunnan@gmail.com (C.-N.Y.); hsujt2813@adm.cgmh.org.tw (J.-T.H.); 3 Graduate Institute of Clinical Medical Sciences, College of Medicine, Chang Gung University, Taoyuan 20401, Taiwan; jonghwei@mail.cgu.edu.tw; 4 Department of Gastroenterology, Chang Gung Memorial Hospital, Chang Gung University, Keelung 20401, Taiwan; leiwei@adm.cgmh.org.tw; 5 Department of Endocrinology and Metabolism, Chang Gung Memorial Hospital, Chang Gung University, Keelung 20401, Taiwan; shengfoung@gmail.com; 6 Faculty of Pharmaceutical Sciences, Teikyo University, Tokyo 13228, Japan; mtakano@pharm.teikyo-u.ac.jp (M.T.); akittaka@pharm.teikyo-u.ac.jp (A.K.); 7 Endocrine Core Lab, Boston University School of Medicine, Boston, MA 02118, USA; taichen@bu.edu; 8 Department of Ophthalmology, Chang Gung Memorial Hospital, Chang Gung University, Keelung 20401, Taiwan; arvin.sun@msa.hinet.net; 9 Department of Anatomy, College of Medicine, Chang Gung University, Taoyuan 20401, Taiwan; 10 Urology Department, Chang Gung Memorial Hospital, Chang Gung University, Taoyuan 20401, Taiwan

**Keywords:** triple negative breast cancer, TNBC, MART-10, EMT, vitamin D

## Abstract

Regarding breast cancer treatment, triple negative breast cancer (TNBC) is a difficult issue. Most TNBC patients die of cancer metastasis. Thus, to develop a new regimen to attenuate TNBC metastatic potential is urgently needed. MART-10 (19-nor-2α-(3-hydroxypropyl)-1α,25(OH)_2_D_3_), the newly-synthesized 1α,25(OH)_2_D_3_ analog, has been shown to be much more potent in cancer growth inhibition than 1α,25(OH)_2_D_3_ and be active *in vivo* without inducing obvious side effect. In this study, we demonstrated that both 1α,25(OH)_2_D_3_ and MART-10 could effectively repress TNBC cells migration and invasion with MART-10 more effective. MART-10 and 1α,25(OH)_2_D_3_ induced cadherin switching (upregulation of E-cadherin and downregulation of N-cadherin) and downregulated P-cadherin expression in MDA-MB-231 cells. The EMT(epithelial mesenchymal transition) process in MDA-MB-231 cells was repressed by MART-10 through inhibiting Zeb1, Zeb2, Slug, and Twist expression. LCN2, one kind of breast cancer metastasis stimulator, was also found for the first time to be repressed by 1α,25(OH)_2_D_3_ and MART-10 in breast cancer cells. Matrix metalloproteinase-9 (MMP-9) activity was also downregulated by MART-10. Furthermore, F-actin synthesis in MDA-MB-231 cells was attenuated as exposure to 1α,25(OH)_2_D_3_ and MART-10. Based on our result, we conclude that MART-10 could effectively inhibit TNBC cells metastatic potential and deserves further investigation as a new regimen to treat TNBC.

## 1. Introduction

Breast cancer, the most common diagnosed cancer and second leading cause of death in women, has about 1 million new cases per year worldwide [1]. With recent great improvement in cancer biology, breast cancer treatment has got a great breakthrough. However, approximately 25% to 50% of breast cancer patients would still develop metastasis eventually, leading to the poor prognosis [2,3].

Triple-negative breast cancer (TNBC) accounts for 12%–20% of all breast cancer and is with more aggressive disease progress and worse prognosis [4,5]. TNBC features the lack of expression of estrogen and progesterone receptors and the lack of overexpression of HER-2, which result in the resistance to anti-hormone therapies and HER-2-aiming target therapies [6]. Since most TNBC patients die of cancer metastasis, finding a new regiment to inhibit TNBC metastasis should be prioritized.

1α,25(OH)_2_D_3_, the active form of vitamin D and originally deemed as only having mineral functions, has been found to have non-classical actions in the late 1970s, such as pro-differentiation, pro-apoptosis, anti-proliferation, anti-invasion, and anti-angiogenesis, leading to the subsequent abundant studies applying vitamin D to treat cancer [7,8,9,10,11]. However, the anti-cancer concentration needed for 1α,25(OH)_2_D_3_ is usually greatly exceeding the physiological concentration, which could induce hypercalcemia as application of 1α,25(OH)_2_D_3_ to treat cancer clinically. To minimize hypercalcemia-inducing effect while maximizing the anti-tumor effect, lots of 1α,25(OH)_2_D_3_ analogs have been synthesized. Regarding breast cancer, *in vitro* studies have shown that 1α,25(OH)_2_D_3_ and its analogs are potent to inhibit breast cancer cell growth [12,13,14,15,16,17]. Nevertheless, there are still no analogs been proven to significantly benefit breast cancer patients in clinical studies [18,19].

One special kind of vitamin D analog with a 19-nor structure (C19 methylene group is replaced by two hydrogen atoms) was synthesized in 1983 [20]. Perlman *et al.* further synthesized 19-nor-1α,25(OH)_2_D_3_ and showed that this kind of analog had similar pro-differentiation and, most importantly, much less calcemia effect as compared to 1α,25(OH)_2_D_3_ [21], which led to the further modification of the A-ring and, thus, generated a variety of 19-nor-viamin D analogs [22], including MART-10. MART-10 (19-nor-2α-(3-hydroxypropyl)-1α,25(OH)_2_D_3_) [23] has been found to be much more potent than 1α,25(OH)_2_D_3_ in inhibiting a variety of cancer cells growth *in vitro* [9,11,24,25,26] and effective to repress pancreatic cancer cell growth *in vitro* and *in vivo* without inducing hypercalcemia [27]. As for cancer metastasis, it has been shown that MART-10 is a promising agent to attenuate pancreatic cancer cell metastatic potential [28]. In terms of breast cancer, MART-10 has been proven to be able to inhibit ER+ MCF-7 cell growth and metastasis [29,30]. In the current study, we aimed to investigate the effect of MART-10 on TNBC metastasis with the attempt to develop a new regimen for TNBC treatment.

## 2. Result

### 2.1. Inhibition of MDA-MB-231 Cell Invasion and Migration by 1α,25(OH)_2_D_3_ and MART-10

**Figure 1:**
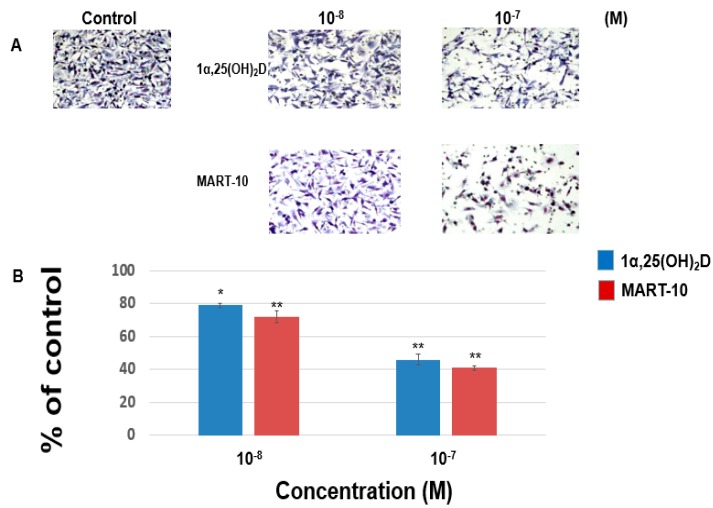
The effects of 1α,25(OH)_2_D_3_ and MART-10 on the migration of MDA-MB-231 cells. The migration of MDA-MB-231 cells pretreated with 1α,25(OH)_2_D_3_ or MART-10 for 48 h was measured by transwell migration assay. Four hours were allowed for cells to migrate and cells were digitally photographed after fixing and staining. Cells that migrated through filters were counted under the microscope (**A**) (IX71, Olympus, Tokyo, Japan). The quantitative result was shown in (**B**). Experiments were performed in triplicate and repeated at least three times (* *p* < 0.05, ** *p* < 0.01).

**Figure 2:**
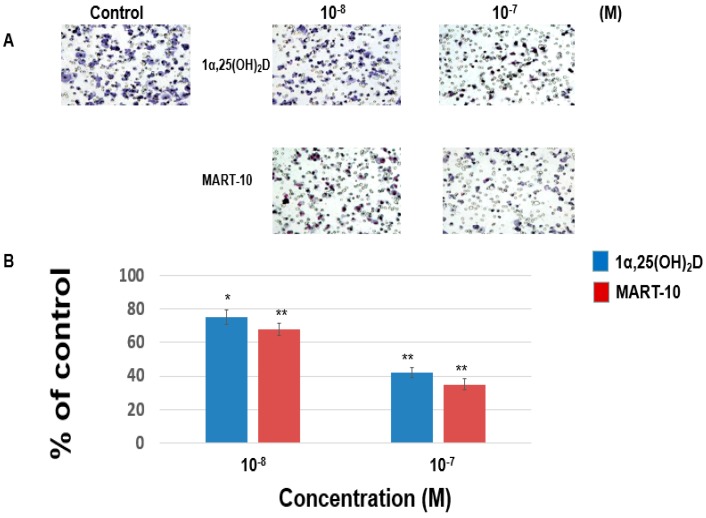
The effects of 1α,25(OH)_2_D_3_ and MART-10 on the invasion of MDA-MB-231 cells. The invasion of MDA-MB-231 pretreated with 1α,25(OH)_2_D_3_ or MART-10 for 48 h was studied by matrigel invasion assay. Twenty-four hours of invasion time were allowed for cells to invade and invading cells were digitally photographed after fixing and staining. Cells that invaded through filters were counted under the microscope (**A**) (IX71, Olympus, Tokyo, Japan). The quantitative result was demonstrated in (**B**). Experiments were performed in triplicate and repeated at least three times (* *p* < 0.05, ** *p* < 0.01).

For cancer cells to metastasis, cells must get abilities to migrate to another location. To achieve this, cancer cells also need the ability to invade the surrounding tissues. Thus, we evaluated the effect of 1α,25(OH)_2_D_3_ and MART-10 on MDA-MB-231 cells migration and invasion. The migration assay (Figure 1) reveals that 1α,25(OH)_2_D_3_ at 10^−8^ and 10^−7^ M inhibited MDA-MB-231 cell migration ability to 79% ± 1.3% and 46% ± 3.4%, as compared to the control. 10^−8^ and 10^−7^ M MART-10 attenuated MDA-MB-231 cell migration ability to 72% ± 3.5% and 41% ± 1.3%. Figure 1 indicates that both 1α,25(OH)_2_D_3_ and MART-10 are potent compounds to inhibit MDA-MB-231 cell migration with MART-10 much more potent than 1α,25(OH)_2_D_3_. The invasion assay (Figure 2) showed that MDA-MB-231 cell invasion ability was repressed to 75% ± 3.7% and 42% ± 3.3% by 10^−8^ and 10^−7^ M 1α,25(OH)_2_D_3_ and 68% ± 4.2% and 35% ± 2.8% by 10^−8^ and 10^−7^ M MART-10. Based on our data, we thus concluded that both 1α,25(OH)_2_D_3_ and MART-10 are effective compounds to inhibit MDA-MB-231 cell metastatic potential with MART-10 more potent than 1α,25(OH)_2_D_3_.

### 2.2. Inhibition of MDA-MB-453 Cell Invasion and Migration by 1α,25(OH)_2_D_3_ and MART-10

**Figure 3:**
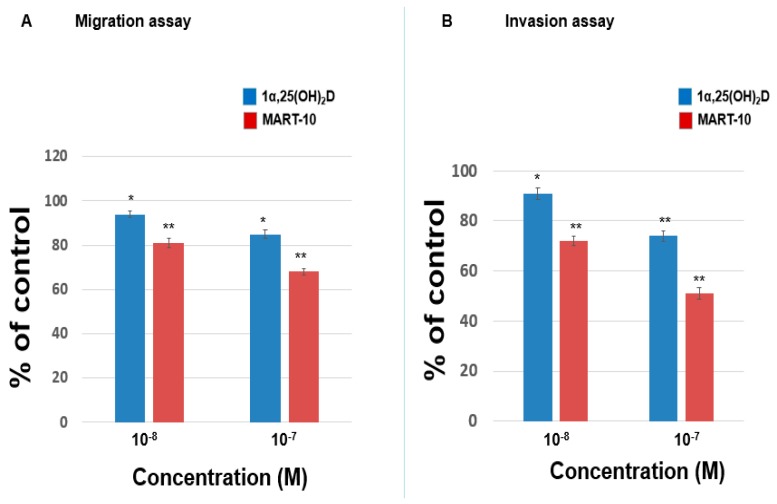
The effects of 1α,25(OH)_2_D_3_ and MART-10 on the migration and invasion of MDA-MB-453 cells. The migration or invasion of MDA-MB-4531 pretreated with 1α,25(OH)_2_D_3_ or MART-10 for 48 h was studied by transwell migration or matrigel invasion assay. Four or 24 h of migration or invasion time were allowed for cells to migrate or invade. The quantitative results were shown in (**A**,**B**) for migration and invasion studes. Experiments were performed in triplicate and repeated at least three times (* *p* < 0.05, ** *p* < 0.01).

Figure 3A shows that 1α,25(OH)_2_D_3_, at 10^−8^ and 10^−7^ M, repressed MDA-MB-453 cells migration ability to 94% ± 1.5% and 85% ± 2% while MART-10, at the same concentration, inhibited MDA-MB-453 cells migration ability to 81% ± 2.1% and 68% ± 1.6%. Figure 3B reveals that the invasion ability of MDA-MB-453 cells was downregulated to 91% ± 2.3% and 74% ± 2.1% by 10^−8^ and 10^−7^ M 1α,25(OH)_2_D_3_, and to 72% ± 1.9% and 51% ± 2.2% by 10^−8^ and 10^−7^ M MART-10. Our result indicates that both MART-10 and 1α,25(OH)_2_D_3_ could significantly attenuate MDA-MB-453 cell metastatic ability and MART-10 is much more effective.

### 2.3. Evaluation of 1α,25(OH)_2_D_3_ and MART-10 Effects on E-, N-, and P-Cadherin of MDA-MB-231 Cells

**Figure 4:**
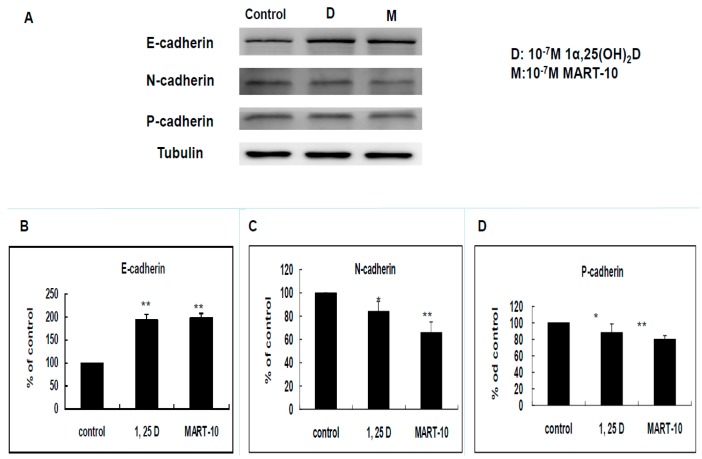
Effects of 1α,25(OH)_2_D_3_ or MART-10 on the expressions of E-cadherin, N-cadherin, and P-cadherin in MDA-MB-231 cells. (**A**) Western blot showing the expressions of E-cadherin, N-cadherin, and P-cadherin in MDA-MB-231 cells after 10^−7^ M 1α,25(OH)_2_D_3_ or MART-10 treatment. Tubulin was used as the loading control; and (**B**–**D**) quantitative analysis of E-cadherin, N-cadherin, and P-cadherin expression levels of MDA-MB-231 cells after two days of 10^−7^ M 1α,25(OH)_2_D_3_ or MART-10 treatment. Each value was a mean ± SD of three independent determinations (* *p* < 0.05, ** *p* < 0.01).

E-cadherin, N-cadherin, and P-cadherin are important cadherins during breast cancer metastasis. The E-cadherin, N-cadherin, and P-cadherin expressions of MDA-MB-231 cells after treatment were determined by Western blot (Figure 4A). As shown in Figure 4B, 10^−7^ M 1α,25(OH)_2_D_3_ and MART-10 upregulated E-cadherin expression to 1.93 ± 0.12 and 1.98 ± 0.07 folds in MDA-MB-231 cells. N-cadherin was repressed to 0.84 ± 0.08 and 0.66 ± 0.09 folds by 10^−7^ M 1α,25(OH)_2_D_3_ and MART-10. Regarding P-cadherin, the expressions was inhibited to 0.88 ± 0.11 and 0.79 ± 0.05 folds by 10^−7^ M 1α,25(OH)_2_D_3_ and MART-10, respectively. Our data indicated that both 1α,25(OH)_2_D_3_ and MART-10 stimulated E-cadherin expression and attenuated N-cadherin and P-cadherin expressions in MDA-MB-231 cells.

### 2.4. Evaluation of 1α,25(OH)_2_D_3_ and MART-10 Effects on the Expression of Epithelial-Mesenchymal Transition (EMT)-Related Transcription Factors, Zeb1 and 2, Snail, Slug, and Twist of MDA-MB-231 Cells

**Figure 5:**
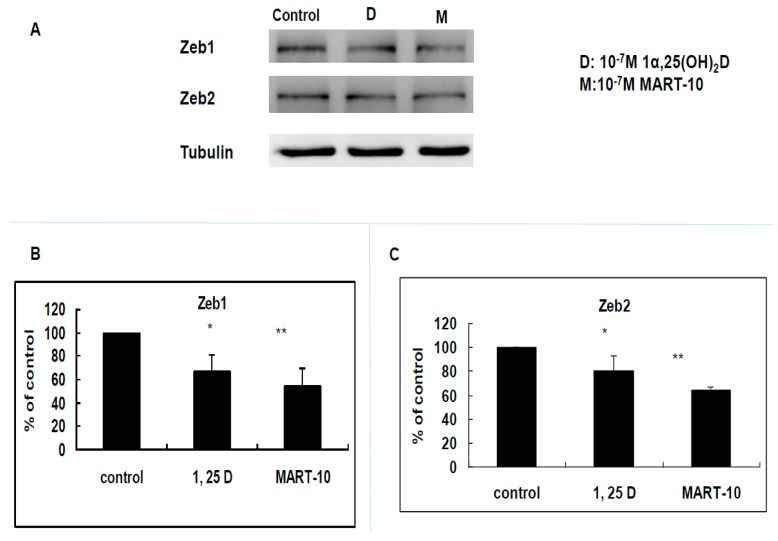
Effects of 1α,25(OH)_2_D_3_ or MART-10 on the expressions of EMT-related transcriptional factors, Zeb1 and Zeb2, in MDA-MB-231 cells. (**A**) Western blot showing the expressions of Zeb1 and Zeb2 in MDA-MB-231 cells after 10^−7^ M 1α,25(OH)_2_D_3_ or MART-10 treatment. Tubulin was used as the loading control; and (**B**,**C**) quantitative analysis of Zeb1 and Zeb2 expression levels of MDA-MB-231 cells after two days of 10^−7^ M 1α,25(OH)_2_D_3_ or MART-10 treatment. Each value was a mean ± SD of three independent determinations (* *p* < 0.05, ** *p* < 0.01).

**Figure 6:**
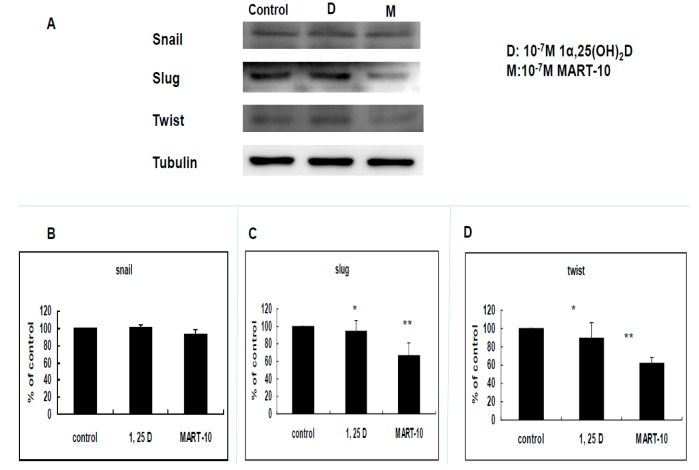
Effects of 1α,25(OH)_2_D_3_ or MART-10 on the expressions of EMT-related transcriptional factors, Snail, Slug, and Twist, in MDA-MB-231 cells. (**A**) Western blot depicting the expressions of Snail, Slug, and Twist in MDA-MB-231 cells after 10^−7^ M 1α,25(OH)_2_D_3_ or MART-10 treatment. Tubulin was used as the loading control; and (**B**–**D**) quantitative analysis of Snail, Slug, and Twist expression levels of MDA-MB-231 cells after two days of 10^−7^ M 1α,25(OH)_2_D_3_ or MART-10 treatment. Each value was a mean ± SD of three independent determinations (* *p* < 0.05, ** *p* < 0.01).

Since EMT plays an important part during cancer metastasis, we next investigated expressions of five transcriptional factors responsible for EMT by Western blot, including Zeb1, and 2, Snail, Slug, and Twist. Figure 5 clearly showed that Zeb1 expression was downregulated to 0.67 ± 0.14 and 0.54 ± 0.15 folds by 10^−7^ M 1α,25(OH)_2_D_3_ and MART-10. The similar phenomenon was observed in Zeb2. 10^−7^ M MART-10 inhibited Slug and Twist expression to 0.67 ± 0.13 and 0.65 ± 0.06 folds while 1α,25(OH)_2_D_3_ at 10^−7^ M downregulated Slug and Twist expression to 0.94 ± 0.13 and 0.9 ± 0.16 folds (Figure 6C,D). As for Snail, neither 1α,25(OH)_2_D_3_ or MART-10 could significantly inhibit its expression in MDA-MB-231 cells (Figure 6B). Collectively, both 1α,25(OH)_2_D_3_ and MART-10 are able to repress Zeb1 and 2, Slug, and Twist expressions in MDA-MB-231 and MART-10 is obviously more potent than 1α,25(OH)_2_D_3_.

### 2.5. Evaluation LCN2 Expression of MDA-MB-231 Cells after 1α,25(OH)_2_D_3_ and MART-10 Treatment

**Figure 7:**
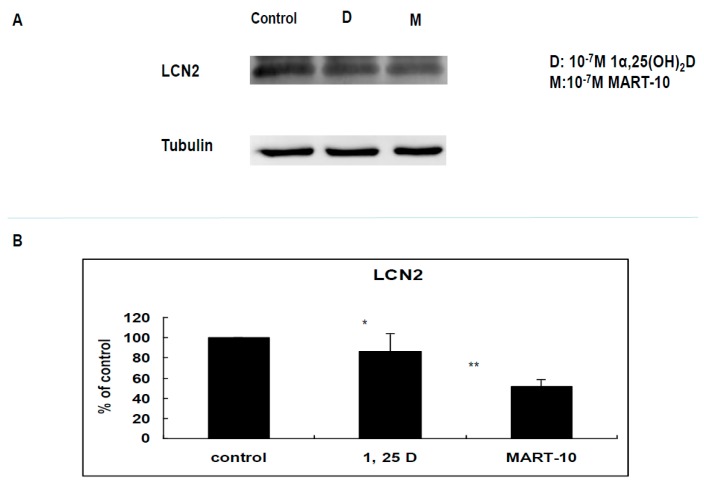
Effects of 1α,25(OH)_2_D_3_ or MART-10 on the expression of LCN2 in MDA-MB-231 cells. (**A**) Western blot depicting the expressions of LCN2 in MDA-MB-231 and cells after 10^−7^ M 1α,25(OH)_2_D_3_ or MART-10 treatment. Tubulin was used as the loading control; and (**B**) quantitative analysis of LCN2 expression levels of MDA-MB-231 cells after two days of 10^−7^ M 1α,25(OH)_2_D_3_ or MART-10 treatment. Each value was a mean ± SD of three independent determinations (* *p* < 0.05, ** *p* < 0.01).

LCN2 has been shown to increase breast cancer invasiveness [31]. We, thus, evaluated the effect of 1α,25(OH)_2_D_3_ and MART-10 on LCN2 expressions of MDA-MB-231 cells. Figure 7B reveals that 10^−7^ M 1α,25(OH)_2_D_3_ or MART-10 inhibited LCN2 expressions to 0.86 ± 0.17 or 0.52 ± 0.06-fold. Our results indicated that MART-10 could effectively repress LCN2 expressions in MDA-MB-231 cells, leading to the attenuation of metastatic ability.

### 2.6. Functional Assay of MMPs by Zymography

**Figure 8:**
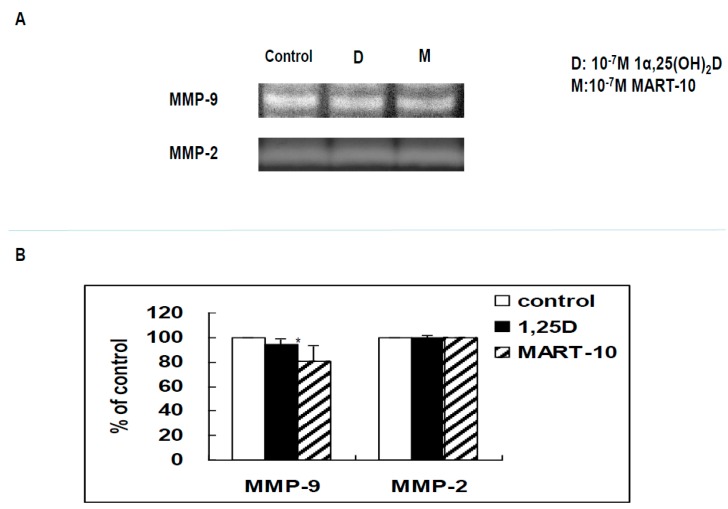
Effects of 1α,25(OH)_2_D_3_ and MART-10 on the activities of MMP-2 and MMP-9 in MDA-MB-231 cells. (**A**) MMP-2 and MMP-9 activity was analyzed by zymography. After two days of treatment with 10^−7^ M 1α,25(OH)_2_D_3_ or MART-10, conditioned media were collected for activity assay. The white bands represented the digested areas corresponding to the activities of MMP-2 and MMP-9; (**B**) Quantitative analysis of MMP-2 and MMP-9 activities. Each value was a mean ± SD of three independent determinations (* *p* < 0.05).

Zymography analysis was further applied to evaluate MMP-2 and MMP-9 activity of MDA-MB-231 cell conditioned media with or without treatment. Figure 8B demonstrates that MART-10 decreased MMP-9, but not MMP-2, activity while 1α,25(OH)_2_D_3_ influenced neither MMP-2 nor MMP-9 activity.

### 2.7. Evaluation of 1α,25(OH)_2_D_3_ and MART-10 Effect on F-Actin Synthesis in MDA-MB-231 Cells

**Figure 9:**
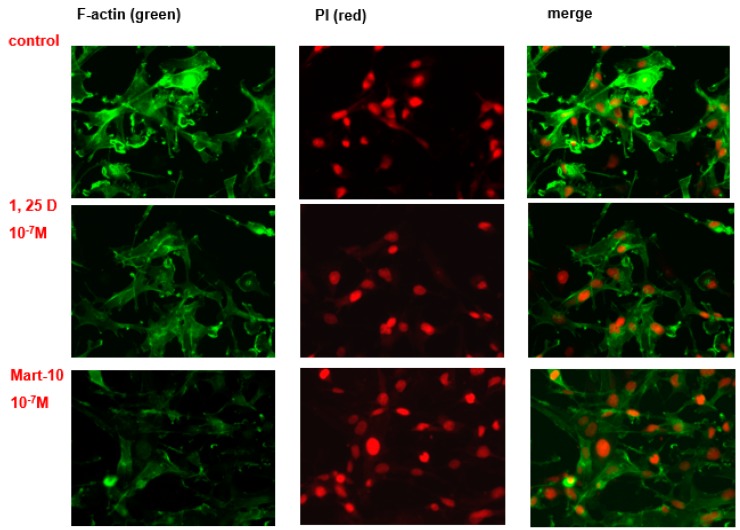
The effects of 1α,25(OH)_2_D_3_ and MART-10 on the formation of F-actin in MDA-MB-231 cells. The expression and cellular distribution of F-actin in MDA-MB-231 cells with or without 1α,25(OH)_2_D_3_ or MART-10 treatment for two days were fluorescently stained by FITC-conjugated phalloidin (green). Propidium iodide (PI) (red) was applied to counter-stain the nucleus.

F-actin plays a vital role in cell migration. MDA-MB-231 cells were double stained with anti F-actin antibody (green) and DAPI (red) for nucleus. The confocal microscope was applied to observe the immunofluorescence intensity and distribution. Figure 9 shows that both 1α,25(OH)_2_D_3_ or MART-10 could effectively reduce MDA-MB-231 cell F-actin synthesis with MART-10 much more than 1α,25(OH)_2_D_3_.

## 3. Discussion

Although great improvement has been achieved for the treatment of primary breast cancer recently, there are still 25% to 50% breast cancer patients who would develop metastasis sooner or later. Due to the limitation of current detective image facility, the detected metastatic lesion is usually growing in the metastatic site and undergoing some gene changes, which may make them more resistant to current available breast cancer therapies. Thus, to develop a new regimen which could inhibit breast cancer growth and repress tumor metastasis is urgently needed, especially for TNBC, which is resistant to adjuvant anti-hormone therapies and target therapies against HER-2.

1α,25(OH)_2_D_3_ exerts its genomic functions through binding with vitamin D receptor (VDR). Liganded VDR would further bind with retinoid X receptor (RXR) to form a heterodimer to bind to vitamin D response element (VDRE) [32], located in the promoter region of vitamin D responsive genes, to influence gene expression. 24-OHase (CYP24A1) is responsible for the degradation of 1α,25(OH)_2_D. Since MART-10 has been proved to have high VDR binding affinity [33] and be more resistant to CYP24A1-mediated degradation [9,25], it is expectable to see MART-10 possesses higher VDR-transactivation as compared to 1α,25(OH)_2_D_3_. In this current study, MART-10 and 1α,25(OH)_2_D_3_ are both shown to be effective in the inhibition of MDA-MB-231 and MDA-MB-453 cell migration and invasion with MART-10 being more potent in these respects.

Cell migration and cell invasion are two important steps for cancer metastasis. Before cancer cells initiate metastasis, they first need to lose cell-cell adhesion. E-Cadherin, a transmembrane protein, is responsible for cell adherence. Loss of E-cadherin-mediated adhesion has been shown to be linked to the neoplastic process, further invasive behaviors [34], as well as poor prognosis [35]. Contrary to E-cadherin, N-cadherin functions as a stimulator of cancer metastasis and growth [36,37,38]. It has been shown that N-cadherin overexpression could increase breast cancer cell invasiveness [39] and severity of pancreatic cancer patients [40]. As shown in Figure 4A–C, both 1α,25(OH)_2_D_3_ and MART-10 increased E-cadherin expression and decreased N-cadherin expression in MDA-MB-231 cells with MART-10 more potent than 1α,25(OH)_2_D_3_. P-cadherin is also one subgroup of cadherins. Overexpression of P-cadherin is linked to high-grade breast cancer. P-cadherin has been demonstrated to be a stimulator of breast cancer cell migration and invasion and a poor prognosis factor of breast cancer patients [41]. Figure 4A,D clearly show that P-cadherin was downregulated by both 1α,25(OH)_2_D_3_ and MART-10. Taken together, we conclude that MART-10 and 1α,25(OH)_2_D_3_ are able to induce cadherin switch (upregulation of E-cadherin and downregulation of N-cadherin) and repress P-cadherin expression in MDA-MB-231 cells, leading to the attenuation of cell invasion and migration shown in Figure 1 and Figure 2.

Epithelial–mesenchymal transition (EMT) is the process of epithelial cell trans-differentiation into mesenchymal cells which have higher motility and invasiveness. EMT is one crucial part amid wound healing, stem cell behaviors, normal development, as well as cancer progression. Regarding cancer treatment, EMT has been shown to make cancer cell obtain stem cell-like properties and resistance to chemotherapy and immune surveillance [42,43,44], thus leading to more aggressive cancer progression and worse outcomes [45]. Three families of transcription factors have been demonstrated to regulate gene expressions responsible for EMT, including Snail/Slug, Zeb1/2, and Twist families [46]. Figure 5 shows that MDA-MB-231 cell Zeb1 and Zeb2 expressions were attenuated by 1α,25(OH)_2_D_3_ and MART-10. MART-10 further repressed Slug and Twist expressions in MDA-MB-231 cells (Figure 6). Moreover, N-cadherin, one of the mesenchymal cell marker [47], is downregulated by both 1α,25(OH)_2_D_3_ and MART-10 (Figure 4A,C). Our data indicate that 1α,25(OH)_2_D_3_ and MART-10 are both able to repress MDA-MB-231 cell EMT process, resulting into the inhibition of metastatic potential shown in Figure 1 and Figure 2.

There is another one important step for cancer metastasis proceeding, which is digestion of extracellular matrix and basement membrane to let cancer cells spread to other sites. MMPs are proteases functioning to digest collagen, thus often found upregulated amid tumor progression [48]. Among others, MMP-9 is the main MMP to digest basement membrane collagen and MMP-2 is found to be positively associated with breast cancer progression [49,50]. Figure 8 demonstrates that 10^−7^ M MART-10 could repress MMP-9, but not MMP-2 activity, while 1α,25(OH)_2_D_3_ had no effect on either MMP-2 or MMP-9 activity in MDA-MB-231 cells.

LCN2 belongs to a member of lipocalin family and has been shown to have pro-proliferation, pro-angiogenesis, as well as pro-metastasis effects in cancer cells [51,52]. Our group has demonstrated LCN2 plays as an oncogene in human cholangiocarcinoa and is also one of the vitamin D responsive genes [53]. As for breast cancer, LCN2 has been demonstrated to increase breast cancer tumorigenesis and metastasis [31]. Figure 7 indicates that LCN2 is also 1α,25(OH)_2_D_3_ responsive gene in MDA-MB-231 cells and MART-10 inhibited LCN2 expression to a greater extent than 1α,25(OH)_2_D_3_ in MDA-MB-231 cells, partly leading to the metastatic potential inhibition noted in Figure 1 and Figure 2.

Filamentous actin (F-actin) cytoskeleton networks function to not only maintain cellular shape but also regulate other important cellular function. In addition, the force generated by F-actin network synthesis is crucial for cell migration [54]. In this study, we observed that F-actin synthesis in MDA-MB-231 cells was repressed by both MART-10 and 1α,25(OH)_2_D_3_ with MART-10 having a more potent effect (Figure 9), resulting into the migration attenuation noted in Figure 1.

Collectively, our result demonstrated that both 1α,25(OH)_2_D_3_ and MART-10 could effectively repress TNBC cell metastasis with MART-10 much more potent than 1α,25(OH)_2_D_3_. The further *in vivo* studies applying MART-10 to treat TNBC should be warranted.

## 4. Material and Method

### 4.1. Vitamin D Compounds

1α,25(OH)_2_D_3_ was purchased from Sigma (St. Louis, MO, USA). 19-nor-2α-(3-hydroxypropyl)-1α,25(OH)_2_D_3_ (MART-10) was synthesized and obtained as previously described [23]. The structures of both drugs were in supplemental data.

### 4.2. Cell Culture

Human breast carcinoma cell lines, MDA-MB-231 and MDA-MB-453, were purchased from BCRC (Hsinchu, Taiwan). MDA-MB-231 and MDA-MB-453 cells were grown in RPMI-1640 (Gibco, Massachusetts, MA, USA) and Leibovitz’s L-15 (Gibco) supplemented with 10% fetal bovine serum (FBS). Culture medium was changed three times per week.

### 4.3. Matrigel Invasion Assay

MDA-MB-231 and MDA-MB-453 cells were pretreated with indicated concentrations of 1α,25(OH)_2_D_3_ or MART-10 for two days. The matrigel invasion assay was performed as previously described [55]. The cells migrated to the opposite side of the matrigel-coated membrane were fixed with 4% paraformaldehyde in 1× PBS, pH 7.5. The number of invaded cells were stained, digitally photographed, and counted under the microscope (IX71, Olympus, Tokyo, Japan). Experiments were performed in triplicates and repeated at least three times.

### 4.4. Trans-Well Filter Migration Assay

MDA-MB-231 and MDA-MB-453 cells which were treated for two days with indicated concentrations of either MART-10 or 1α,25(OH)_2_D_3_ were seeded on each trans-well filter with 8.0-μm pores (Costar, Cambridge, MA, USA). The procedure was performed as previously described [30]. The migrated cells on the lower surface of the filter were stained and counted under four random high-power microscopic fields (HPF; 100×) per filter, and the mean number of cells that migrated through the filter was calculated for each condition. The experiments were performed in triplicates.

### 4.5. Gelatin Zymography

The detailed procedures were described previously [30]. To analyze the gelatinolytic proteins in conditioned media of cultured cells treated by either MART-10 or 1α,25(OH)_2_D_3_ for two days, samples were run under non-reducing conditions in 10% SDS-polyacrylamide gels containing 2 mg/mL gelatin and MMP activities in the gel were assayed overnight in reaction buffer at 37 °C.

### 4.6. Western Blot

After two days of treatment with 10^−7^ M MART-10 or 1α,25(OH)_2_D_3_, cells were washed once with PBS and lyzed in the lysis buffer containing 50 mM Tris-HCl, 50 mM β-glycerol phosphate, 50 mM NaCl, 1 mM Na_3_Vo_4_, 1 mM EDTA, 1 mM EGTA, 1% NP40, and freshly added 1 mM DTT, 1 mM PMSF, 2 μg/mL aprodenin, 2 μg/mL leupeptin, and 2 μg/mL pepstatin right before lysis. The antibodies used in this experiment were monoclonal antibodies against E-cadherin (1:1000, #3195, Cell Signaling Technology, Irvine, CA, USA), N-cadherin (1:1000, #13116, Cell Signaling Technology), P-cadherin (1:1000, #2189, Cell Signaling Technology), Zeb1 (1:500, TA802313, OriGene Technologies, Inc., Rockville, MD, USA), Zeb 2 (1:500, TA802113, OriGene Technologies), Snail (1:100, PA5-23472, Thermo Fisher Scientific, Waltham, MA, USA), Slug (1:1000, #9585, Cell Signaling Technology), Twist (1:100, sc-15393, Finnell Street Dallas, TX, USA), LCN2 (1:500, PAB9543, Abnova, Taipei, Taiwan). After washing in TBST, the blots were detected using ECL reagents (Millipore, WBKLS0500, Temecula, CA, USA). The second body was goat anti-mouse/rabbit IgG conjugated with HRP (Cell Signaling Technology, Boston, MA, USA). The incubation time for primary or secondary antibody was 2 or 1 h, respectively, under room temperature. 10% SDS-PAGE and Tris-Glycine buffer system were applied. PVDF membrane and wet transfer (400 mA, 2 h) were used. Membranes were detected and analyzed by VersaDoc™ Imaging System (Bio-Rad, Hercules, CA, USA). Expression of targeted protein relative to tubulin (as the loading control) was calculated. The detailed procedure was described previously [30].

### 4.7. F-Actin Staining

MDA-MB-231 cells were seeded on glass coverslips in cultured dish and allowed to attach overnight. After two days of treatment with either MART-10 or 1α,25(OH)_2_D_3_, cells were fixed with 4% paraformaldehyde in 1× PBS, pH 7.5, at room temperature. The F-actin protein was revealed by incubation with FITC-conjugated phalloidin and examined using confocal microscope (LSM510 Meta, Zeiss, Oberkochen, Germany).

### 4.8. Statistics Method

The data from each group were compared by the student *t-*test. *p-*value <0.05 was considered as a significant difference. The program of Excel 2010 (Microsoft, Washington, DC, USA) was employed to conduct the statistics.

## 5. Conclusions

Even with current significant progress in breast cancer treatment, 25% to 50% of breast cancers would eventually develop metastasis, leading to poor prognosis. TNBC belongs to a special subtype of breast cancer with more aggressive disease progression and worse prognosis. Since anti-hormone treatment and HER-2-targeting therapies are not suitable for TNBC, to develop a new regiment against TNBC should be warranted. In this current study, we showed that both 1α,25(OH)_2_D_3_ and MART-10 could effectively attenuate triple negative breast cancer cells metastatic potential through repression of EMT process and induction of cadherin switching (upregulation of E-cadherin and downregulation of N-cadherin) with MART-10 much more potent than 1α,25(OH)_2_D_3_. Both drugs further inhibited p-cadherin, and LCN2 expressions in MDA-MB-231 cells. MART-10 also repressed MMP-9 activity. Since MART-10 have been shown to be active in inhibition of cancer growth without inducing obvious side effects *in vivo* [27], further *in vivo* studies regarding the application of MART-10 to treat TNBC is warranted.

## Supplementary Materials

Supplementary materials can be found at http://www.mdpi.com/1422-0067/17/4/606/s1.
