# Supplementary material for: The Vitamin D Analog, MART-10, Attenuates Triple Negative Breast Cancer Cells Metastatic Potential
Source: Int J Mol Sci. 2016 Apr 21;17(4):606. doi: 10.3390/ijms17040606 (PMC4849057; doi:10.3390/ijms17040606)
Supplement: Supplementary file 1 [file ijms-17-00606-s001.pdf]

Supplementary Materials: The Vitamin D Analog, MART-10, Attenuates Triple Negative Breast Cancer Cells Metastatic Potential

Kun-Chun Chiang, Ta-Sen Yeh, Shin-Cheh Chen, Jong-Hwei S. Pang, Chun-Nan Yeh, Jun-Te Hsu, Li-Wei Chen, Sheng-Fong Kuo, Masashi Takano, Atsushi Kittaka, Tai C. Chen, Chi-Chin Sun and Horng-Heng Juang

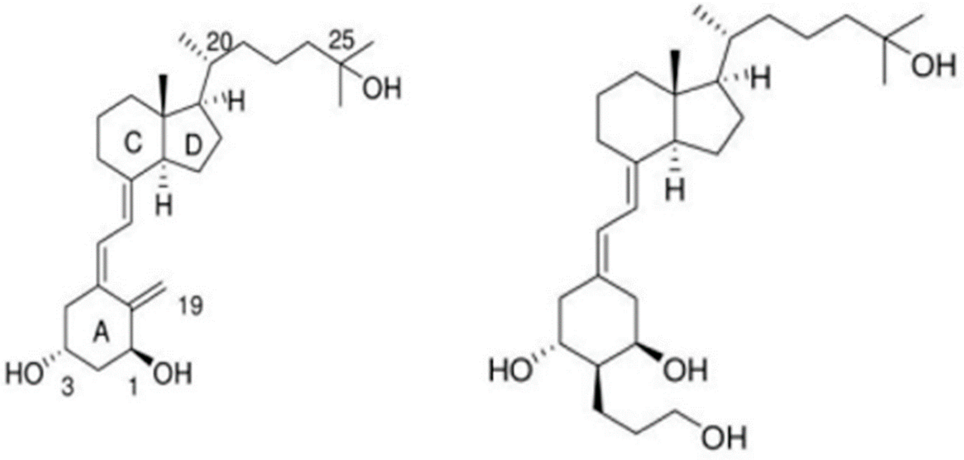

$1\alpha,25(OH)_2D_3$ MART-10

Figure S1. Structure for 1α,25(OH)$_2$D$_3$ and MART-10.
